# Cataract surgery-related complications in patients with end-stage renal disease- a nationwide population-based study in Taiwan

**DOI:** 10.1038/s41598-020-59160-7

**Published:** 2020-02-07

**Authors:** Ching-Hsing Hsiao, Fu-Wen Liang, Chung-Han Ho, Yi-Chen Chen, Jhi-Joung Wang, Chung-Hsi Hsing, Chia-Chun Wu

**Affiliations:** 1 Department of Ophthalmology, Chi Mei Medical Center, Chia Li, Tainan City, Taiwan; 2 Department of Public Health, Kaohsiung Medical University, College of Health Sciences, Kaohsiung, Taiwan; 3 Department of Medical Research, Chi Mei Medical Center, Tainan City, Taiwan; 4 Department of Hospital and Health Care Administration, Chia Nan University of Pharmacy and Science, Tainan City, Taiwan; 5 Department of Anesthesiology, Chi Mei Medical Center, Tainan City, Taiwan; 6 Department of Nephrology, Chi Mei Medical Center, Tainan City, 701 Taiwan; 7 Department of Pharmacy, Chia Nan University of Pharmacy and Science, Tainan City, Taiwan; 8 AI Biomed Center, Southern Taiwan University of Science and Technology, Tainan City, Taiwan; 9 Department of Medical Research, Kaohsiung Medical University Hospital, Kaohsiung, Taiwan; 10 Research Center for Environmental Medicine, Kaohsiung Medical University, Kaohsiung, Taiwan

**Keywords:** Eye diseases, Nephrology

## Abstract

This nationwide retrospective case-control study was aimed at elucidating the risk from cataract surgery in end-stage renal disease (ESRD) patients. Cataract surgery patients were identified using the diagnostic and procedural codes for International Classification of Diseases, 9^th^ Revision, Clinical Modification from Taiwan’s National Health Insurance Research Database. ESRD patients were selected as cases, while propensity scores for age, sex, comorbidities and year-of-surgery-matched patients without chronic kidney disease constituted the controls. Patients who had undergone eye surgery within 3 years before cataract surgery were excluded. The main outcome measures were target cataract surgery-related complications within 3 months after surgery. A total of 352 cases and 1,760 controls were analysed. Patients with ESRD had a 5.06-fold (95% CI: 2.36–10.87; p < 0.001) risk of vitreous haemorrhage and a 2.74-fold (95% CI: 1.20–6.27; p = 0.017) risk of re-operation for dropped nucleus or vitreous complications. Non-diabetic ESRD patients had a 3.49-fold (95% CI: 1.36–8.91; *p* = 0.009) risk of corneal oedema. In conclusion, ESRD patients have a higher risk of vitreous haemorrhage, re-operation for dropped nucleus or vitreous complications and corneal oedema (non-diabetic patients) after cataract surgery. Pre-surgery corneal examination, surgery procedure and medication adjustment, closer and longer post-surgery follow-up may lower the risk and improve the visual outcome.

## Introduction

Cataract is the most common cause of blindness worldwide, and successful cataract surgery restores vision to most patients. Although cataract surgery is a minor procedure, complications sometimes develop that influence the visual outcome. In particular, cataract surgery can lead to glaucoma, vitreous haemorrhage (VH), retinal detachment, intraocular dislocation, dropped nucleus, and wound dehiscence^1^.

Patients with end-stage renal disease (ESRD) were reported to have an increased risk of complications such as bleeding, infection and perioperative mortality when they received major inpatient surgeries^2–4^. ESRD patients were noted to have a higher risk of cataract^5^. Only a few studies have reported cataract surgery-related risk in ESRD patients^6,7^, and these studies involved only a small number of patients and no control group.

The purpose of the present study was to elucidate the risk of cataract surgery-related complications in patients receiving dialysis and to compare this risk to those in patients without chronic kidney disease (CKD) or ESRD. As such, the study may inform better patient care and improve surgical outcomes.

## Methods

### Data source

This retrospective, case-control study used the Longitudinal Health Insurance Database 2000 (LHID2000), which is a subset database of the Taiwan National Health Insurance Research Database (NHIRD) provided by the Bureau of National Health Insurance of the Ministry of Health and Welfare (http://nhird.nhri.org.tw/en/Data_Subsets.html). Taiwan’s National Health Insurance program currently covers more than 23 million people, representing more than 99% of the population of Taiwan. The LHID2000 was a longitudinal dataset randomly selected sample of one million health insurance beneficiaries (4.5% of Taiwan’s population) from the original NHIRD in 2000 to Dec-2013. The sample was not significantly different from the entire population in terms of age, sex, or average insured payroll-related amount. All diseases of both inpatients and outpatients were identified using the International Classification of Diseases, 9^th^ Revision, Clinical Modification (ICD-9-CM) diagnostic and procedural codes. NHIRD is released to the public for research purposes; patient identification numbers and other sensitive personal data have been encrypted to protect individual privacy. The Institutional Review Board (IRB) of Chi Mei medical centre approved this study and waived the requirement for patients’ informed consent as part of the study approval (IRB number: 10709 –E02).

### Inclusion criteria, exclusion criteria and study design

The selected participants were patients in the LHID2000 with diagnosis records of cataract (ICD-9-CM: 366.1, 366.2, 366.3, and 366.9) who had submitted medical expenditure applications for cataract removal surgery (86007C, 86008C, 86009C, and 86010B) between January 1, 2002, and December 31, 2013. To avoid potential biasing of the incidence of complications, patients who had undergone the following eye surgery procedures within 3 years before or at the same day of cataract surgery were excluded: removal of either implanted lens or pseudophakos (ICD-9-OP: 13.8), other lens operations (ICD-9-OP: 13.9), removal of foreign body from the posterior segment of the eye (ICD-9-OP: 14.0), repair of retinal detachment with scleral buckling and implant (ICD-9-OP: 14.4), other repair of retinal detachment, including that with drainage (ICD-9-OP: 14.5), removal of surgically implanted material from the posterior segment of eye (ICD-9-OP: 14.6), operations on the vitreous (ICD-9-OP: 14.7). Patients with a history of any CKD diagnosis (ICD-9-CM: 016.0, 095.4, 042, 223, 236.9, 250.4, 271.4, 274.1, 403, 404, 440.1, 442.1, 447.3, 446.21, 581, 583, 585–589, 591, 593, and 753) but not on regular dialysis and patients with history of glaucoma (ICD-9-CM: 365) were also excluded.

The cases group was defined as those patients with ESRD (ICD-9 code: 585) who had been receiving either HD or PD for more than 3 months and had both catastrophic illness certification and records of medical expenditure applications. In Taiwan, patients with the certificated catastrophic illness, including ESRD, could get care for the illness or related conditions without co-payment for outpatient or inpatient care under Department of Health and Welfare guidelines via physician diagnosis. The records of medical expenditure applications were used to identify the HD (58001C, 58008C, 58027C, 58029C, 58030B) or PD (58002C, 58011C, 58017C, 58028C, 58009B, 58010B, 58012B).

**Figure 1:**
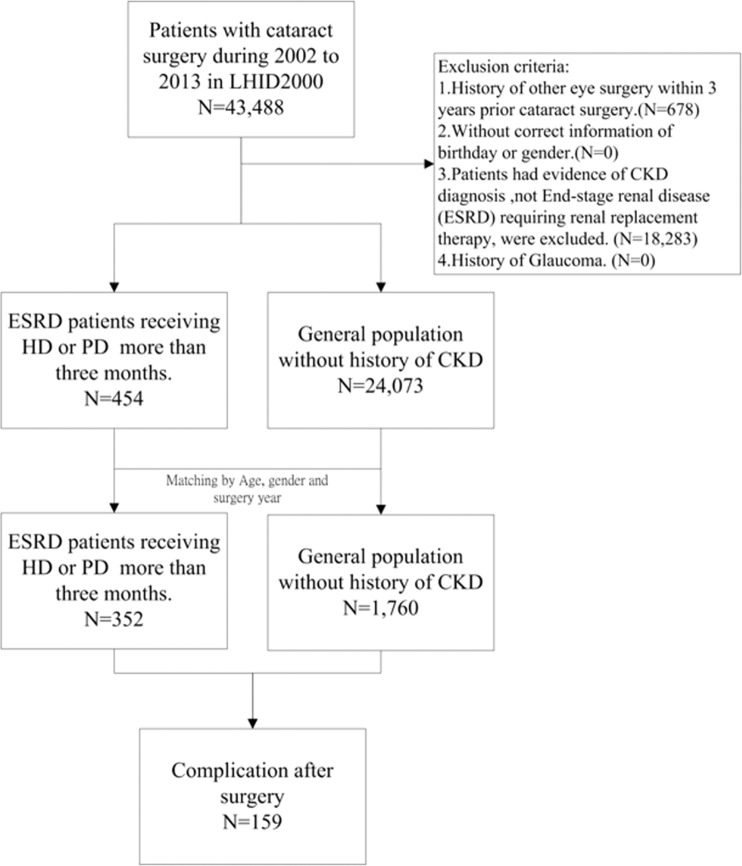
Flowchart of study design. CKD, chronic kidney disease; ESRD, end-stage renal disease; HD, haemodialysis; PD, peritoneal dialysis.

For reducing the potential bias and understanding the individual effect of PD and HD on surgery outcome, patients who underwent both HD and PD treatments were not included. In addition, the following variables were considered as adjustment factors: hospital level, history of antiplatelet drug/anticoagulants or steroid use and B-scan ultrasonography (B-scan) examination Controls were defined as those patients undergoing cataract surgery who were not receiving dialysis therapy. One to five propensity score matching was used to reduce the potential selection bias. The propensity score was estimated from a multivariable logistic regression model with age, sex, year of surgery (+/−1 year), and comorbidities within 1 year before cataract surgery, namely HTN (ICD-9-CM: 401–405, 437.2, 362.11), DM (ICD-9-CM: 250), and myopia (ICD-9-CM: 367), as covariates. This approach could consider the baseline differences between cases and controls^8,9^. Figure 1 shows the flow chart of study subject selection.

### Outcome measures

The outcomes of interest were complications that occurred within 3 months after the cataract surgery, identified by ICD-9 diagnosis codes that may require hospitalisation or surgical intervention, namely glaucoma (ICD-9-CM: 365), endophthalmitis (ICD-9-CM: 360.0, 988.59, 996.69), vitreous haemorrhage (VH) (ICD-9-CM: 379.23) and corneal oedema (372.10), and by ICD-9 procedure codes which were cataract reoperation-related complications: retinal detachment (ICD-9-OP: 14.3–14.5, 14.9), intraocular lens dislocation (ICD-9-OP: 13.70, 13.72, 13.8), dropped nucleus or vitreous complications (ICD-9-OP: 13.9, 14.70–14.79) and wound dehiscence (ICD-9-OP: 11.5×, 12.83, 12.66).These complications definitions are adapted from previous studies with modification^10,11^.

### Statistical analyses

Categorical variables are presented as frequencies with percentages, and differences between the case and control groups were evaluated using the chi-square test. The logistic regression with Firth’s penalised likelihood approach was used to estimate the association risk of complications between the ESRD patients and controls for initial analysis. Considering the potential effects of multiple confounding factors to estimate the association, the disease risk scores (DRS) was used to make the compared group with similar underlying diseases^12,13^. In addition, for matched controls, conditional logistic regression using Firth’s penalised likelihood approach was used to estimate the ORs and 95% CIs of all complications of interest between the case and control groups. To estimate the bias of rare outcome parameters, we used Firth’s penalised likelihood approach^14,15^. In addition, the stratified analysis was performed to evaluate the effect of DM and HTN. P-values < 0.05 were considered statistically significant. SAS 9.4 for Windows software (SAS Institute, Cary, NC, USA) was used to carry out all the analyses.

### Ethics approval and consent to participate

The Research Ethics Committee of Chi Mei Medical Center approved this study and waived the requirement for patients’ informed consent as part of the study approval (IRB No.: 10709–E02).

## Results

In total, 43,488 patients received cataract surgery during the 2000–2013 period; 18,961 patients were excluded according to the criteria. In the remaining 24,527 patients, 352 with ESRD were identified as the case group, and 1,760 patients with matching age, sex, and year of surgery were selected as the control group. Figure 1 shows a flow chart of the study. The mean ages of the patients were 67.25 ± 9.78 and 66.93 ± 9.7 in the case and control groups respectively. About 98.3% patients in the ESRD group received haemodialysis (HD) as renal replacement therapy. The percentage of hypertension (HTN), diabetes mellitus (DM) and myopia in the ESRD group was similar to the matched control group. Compared to the matched controls, a higher percentage of patients in the ESRD group received cataract surgery in a medical centre and less at a local clinic. Antiplatelet drugs/anticoagulants had been prescribed within one month before surgery to treat 17/352 (4.8%) patients in the ESRD group and 114/1760 (6.5%) patients in the control group (P = 0.24). Five (1.42%) in the ESRD group and 38 (2.16%) in the control group ever had either oral or intravenous steroid treatment within 3 months before surgery date. A significant higher rate of B-scan was performed in the ESRD group (15/352, 4.26%) than in the control group (38/1760, 2.16%). We also compared the two groups before matching; the patients in the ESRD group had a higher rate of HTN, DM, myopia, surgery in a medical centre and receiving B-scan examination than Control had. Table 1 shows the other characteristics of the study patients.

**Table 1:** Baseline characteristics and comparisons between the ESRD and Controls.

	Before matching			After matching		
	ESRD patients N = 454	Controls N = 24073	P-value	ESRD patients N = 352	Controls N = 1760	P-value
Age, Mean ± SD	66.40 ± 10.21	69.49 ± 10.03	<0.0001	67.25 ± 9.78	66.93 ± 9.70	0.5674
**Age groups, n(%)**						
55<	53 (11.67)	1746 (7.25)	<0.0001	33 (9.38)	141 (8.01)	0.5486
55–65	149 (32.82)	5198 (21.59)		113 (32.10)	590 (33.52)	
65–75	151 (33.26)	9820 (40.79)		122 (34.66)	645 (36.65)	
75–85	89 (19.60)	6435 (26.73)		74 (21.02)	353 (20.06)	
≧85	12 (2.64)	874 (3.63)		10 (2.84)	31 (1.76)	
**Gender n(%)**						
Female	230 (50.66)	13847 (57.52)	0.0034	186 (52.84)	1011 (57.44)	0.1117
Male	224 (49.34)	10226 (42.48)		166 (47.16)	749 (42.56)	
**Dialysis n(%)**						
HD	447 (98.46)			346 (98.30)		
PD	7 (1.54)			6 (1.70)		
**Comorbidity n(%)**						
HTN	345 (75.99)	10986 (45.64)	<0.0001	269 (76.42)	1359 (77.22)	0.7458
DM	297 (65.42)	4632 (19.24)	<0.0001	216 (61.36)	1051 (59.72)	0.5646
Myopia	25 (5.51)	2474 (10.28)	0.0009	22 (6.25)	88 (5.00)	0.3353
**Hospital level n(%)**						
Centre	96 (21.15)	3450 (14.33)	<0.0001	73 (20.74)	216 (12.27)	<0.0001
Regional	83 (18.28)	3162 (13.14)		63 (17.90)	261 (14.83)	
Local	275 (60.57)	17461 (72.53)		216 (61.36)	1283 (72.90)	
Antiplatelets or Anticoagulants n(%)	22 (4.85)	1006 (4.18)	0.4824	17 (4.83)	114 (6.48)	0.2420
Prolonged steroid treatment n(%)	7 (1.54)	448 (1.86)	0.6176	5 (1.42)	38 (2.16)	0.3704
B-SCAN n(%)	23 (5.07)	403 (1.67)	<0.0001	15 (4.26)	38 (2.16)	0.0213

Abbreviations: DM, diabetes mellitus; ESRD, end-stage renal disease; HD, haemodialysis; HTN, hypertension; PD, peritoneal dialysis; SD, standard deviation.

Under propensity score matching analysis, the most common cataract surgery-related complications of interest were VH (15 [4.26%]) in the ESRD group and glaucoma (55 [3.13%]) in the control group. The second most common complication was corneal oedema and glaucoma in the ESRD group (both being 12 [3.41%]) and corneal oedema in the control group (41 [2.33%]). Patients in ESRD group had a significant higher rate to have a VH (15 [4.26%] vs 15 [0.85%] with an odds ratio (OR) of 5.06, 95% Confidence Interval (CI): 2.36–10.87; p < 0.001) and a higher rate to have a re-operation for dropped nucleus or vitreous complications (10 [2.84%] vs 16 [0.91%] with the OR of 2.74, 95% CI: 1.20–6.27; p = 0.017). The majority of re-operation was for vitreous complications with 10/10 in the ESRD group and 13/16 in the control group. Other complications rarely occurred with a rate less than one percent. (Table 2) Corneal oedema usually happened in the first month in both groups, but with a 9.42 folds increased risk (95% CI: 1.68–52.77) in the ESRD group compared with Controls in the second month. Most VH developed within 2 months and the risk was significantly higher in the ESRD group in the first month (OR 4.39; 95% CI: 1.55–12.4); the second month (OR 5.91; 95% CI: 1.75–20.03) and even the third month (OR 5.08; 95% CI: 0.21–123.16) after surgery. The re-operation for dropped nucleus or vitreous complications developed more commonly (8/10) in the second month in the ESRD group with a 5.53-times higher risk than that in the control group. (Table 3) When we used the Disease Risk score (DRS) to analyse the risk, the most common complication was glaucoma in both groups and followed by VH in the ESRD and corneal oedema in the Controls. The ESRD group had a higher risk of VH (OR 5.76; 95% CI: 3.42–9.71) and surgery for dropped nucleus or vitreous complications (OR 2.65; 95% CI: 1.38–5.08), which was compatible with the results using propensity score matching analysis.

**Table 2:** The multiple complications after cataract surgery in the ESRD and Controls.

	Before matching				After matching			
	ESRD patients n (%) n = 454	Controls n (%) n = 24073	Adjusted OR^a^ (95% CI)	P-value	ESRD patients n (%) n = 352	Controls n (%) n = 1760	Adjusted OR^b^ (95% CI)	P-value
Corneal oedema	14 (3.08)	484 (2.01)	1.64 (0.96–2.81)	0.0711	12 (3.41)	41 (2.33)	1.57 (0.81–3.05)	0.1829
Glaucoma	40 (8.81)	2126 (8.83)	0.81 (0.58–1.13)	0.2135	12 (3.41)	55 (3.13)	1.07 (0.56–2.05)	0.8301
Endophthalmitis	1 (0.22)	81 (0.34)	0.89 (0.18–4.38)	0.8828	1 (0.28)	7 (0.40)	0.62 (0.07–5.17)	0.6544
VH	20 (4.41)	97 (0.48)	5.76 (3.42–9.71)	<0.0001	15 (4.26)	15 (0.85)	5.06 (2.36–10.87)	<0.0001
Retinal detachment	3 (0.66)	64 (0.27)	1.98 (0.66–5.93)	0.2204	3 (0.85)	5 (0.28)	3.21 (0.68–15.24)	0.1428
Intraocular lens dislocation	2 (0.44)	43 (0.18)	2.14 (0.60–7.63)	0.2426	2 (0.57)	2 (0.11)	4.90 (0.65–36.72)	0.1218
Dropped nucleus	10 (2.20)	157 (0.65)	2.65 (1.38–5.08)	0.0034	10 (2.84)	16 (0.91)	2.74 (1.20–6.27)	0.0170
Wound dehiscence	1 (0.22)	26 (0.11)	1.64 (0.33–8.14)	0.5481	1 (0.28)	2 (0.11)	3.26 (0.26–40.27)	0.3565

Abbreviations: CI, confidence interval; ESRD, end stage renal disease; OR, odds ratio; VH, vitreous haemorrhage.

^a^Adjusted by the summary of confounding variables in a single disease risk scores.

^b^Adjusted by hospital level and use of antiplatelets or anticoagulants and according to the sample sizes, we used the Firth’s penalized likelihood approach.

**Table 3:** Major cataract surgery-related complications at different time points.

	1^st^ month			2^nd^ month			3^rd^ month		
	Number of events		Adjusted OR^a^ (95% CI)	Number of events		Adjusted OR^a^ (95% CI)	Number of events		Adjusted OR^a^ (95% CI)
	ESRD	Controls		ESRD	Controls		ESRD	Controls	
Corneal oedema	8	39	1.10 (0.50–2.39)	4	2	9.42* (1.68–52.77)	0	0	—
Glaucoma	11	45	1.21 (0.61–2.39)	1	7	0.59 (0.06–5.39)	0	3	—
VH	7	9	4.39* (1.55–12.40)	7	5	5.91* (1.75–20.03)	1	1	5.08* (0.21–123.16)
Surgery for dropped nucleus or vitreous complications	1	7	0.51 (0.06–4.44)	8	7	5.53* (1.92–15.93)	1	2	1.93 (0.11–33.02)

Abbreviations: CI, confidence interval; ESRD, end stage renal disease; OR, odds ratio; VH, vitreous haemorrhage.

^a^Adjusted by hospital level and use of antiplatelets or anticoagulants.

*P-value < 0.05.

Because DM and HTN are important risk factors for cataract surgery related complications, we performed the stratified analysis. Patients who have both DM and ESRD carried a significantly higher risk of VH than patients who have DM but no CKD/ESRD (OR 4.51; 95% CI: 1.98–10.28). Patients who were non-diabetic ESRD had a significantly higher risk of corneal oedema (OR 3.45; 95% CI: 1.36–8.91) and borderline increased risk of re-operation for dropped nucleus or vitreous complications (OR 5.24; 95% CI: 0.97–28.37) than patients without DM and CKD/ESRD. (Table 4).

**Table 4:** Stratification analysis in patients with and without DM or HTN.

DM status											
	Patients without DM.						Patients with DM.				
	Number of events			Adjuster OR^a^ (95% CI)	P-value		Number of events			Adjuster OR^a^ (95% CI)	P-value
	ESRD patients N = 136	Controls N = 709					ESRD patients N = 216		Controls N = 1051		
Corneal oedema	8	12		3.49 (1.36–8.91)	0.0091		4		29	0.74 (0.26–2.17)	0.5868
Glaucoma	6	16		1.85 (0.67–5.08)	0.2298		6		39	0.74 (0.31–1.78)	0.4981
VH	2	1		5.39 (0.22–131.37)	0.3009		13		14	4.51 (1.98–10.28)	0.0003
Dropped nucleus	3	3		5.24 (0.97–28.37)	0.0546		7		13	2.37 (0.89–6.32)	0.0843
**HTN status**											
	**Patients without HTN**							**Patients with HTN**			
	**Number of events**		**Adjuster OR**^**a**^ **(95% CI)**			**P-value**		**Number of events**		**Adjuster OR**^**a**^ **(95% CI)**	**P-value**
	**ESRD patients N = 83**	**Controls N = 401**						**ESRD patients N = 269**	**Controls N = 1359**		
Corneal oedema	1	5	1.16 (0.13–10.34)			0.8930		11	36	1.61 (0.80–3.23)	0.1843
Glaucoma	1	11	0.37 (0.04–3.19)			0.3672		11	44	1.27 (0.64–2.52)	0.4941
VH	3	1	12.81 (0.89–185.28)			0.0614		12	14	4.15 (1.79–9.63)	0.0009
Dropped nucleus	1	5	0.16 (0.01–4.00)			0.2609		9	11	3.89 (1.55–9.76)	0.0037

Abbreviations: CI, confidence interval; DM, diabetes mellitus; ESRD, end-stage renal disease; HTN, hypertension; OR, odds ratio; VH, vitreous haemorrhage.

^a^Adjusted by all the risk factors in Table 1.

## Discussion

In the present study, we found that patients with ESRD had a higher risk of VH and re-operation for dropped nucleus or vitreous complications after cataract surgery than patients without ESRD after adjusting for the use of antiplatelet drugs/anticoagulants and hospital level. The increased VH risk was apparent through the three months after surgery and the increased re-operation for dropped nucleus or vitreous complications was only evident in the second month. Both non-diabetic and diabetic patients with ESRD have a higher risk of cataract surgery-related complications: a higher risk of corneal oedema and re-operation for dropped nucleus or vitreous complications in non-diabetic patients and a higher risk of VH in diabetic patients.

This study is the first case-control cohort study to focus on cataract surgery-related complications in ESRD patients. There were two publications of cataract surgeries in ESRD patients. Dursun *et al*.^6^ described the surgery-related complications in 18 eyes of ESRD patients without control group design. Luo *et al*.^7^ reported that the surgery-related complications were more common in the ESRD patients than the transplant patients at one week after surgery, but no statistical analyses were performed.

Our findings of increased risk of VH and re-operation for dropped nucleus or vitreous complications after cataract surgery in ESRD patients are perhaps because of a change in the eye’s structure, because of dynamic changes of the eyes during HD, or because a tendency towards bleeding. Another finding of higher corneal oedema after cataract surgery in non-diabetic ESRD patients might be explained by the structural alterations of corneal endothelial cells (EC) that are more susceptible to damage.

VH is the extravasation of blood into space within or near the vitreous body. It can be caused by retinal trauma, abnormal retinal neovascularization, and bleeding from other eye structures due to age-related macular degeneration or choroidal melanoma. In particular, abnormal retinal neovascularisation can occur as a result of diabetic retinopathy, retinal vein occlusion and other rare diseases. Several studies have shown a close association between retinal disorders and CKD in hypertensive patients both with and without DM^16–18^. The Chronic Renal Insufficiency Cohort study reported that the severity of retinopathy was correlated with the level of CKD^19^. This correlation may have arisen because the same microvascular damage process causes both retinopathy and nephropathy. Besides, the higher rate of retinal vein occlusion and age-related macular degeneration in ESRD patients may also contribute to the increased risk of VH after cataract surgery^20,21^.

Alterations to the composition of vitreous humour in pseudophakic eyes after cataract surgery was discovered by Neal *et al*.^22^ and they suggested that these alterations may contribute to the retinal complications after cataract surgery. In ESRD patients, changes in intraocular pressure (IOP)^23–25^ and ocular structures^26,27^ during HD were reported in different studies. The ocular structure changes include decrease in choroidal thickness and retina thickness after HD. The changes of colloid osmotic pressure caused by uraemia toxin and fluid removal during HD was considered the explanation for intradialytic IOP and ocular structure changes, which may predispose ESRD patient to have VH after cataract surgery.

The bleeding tendency in patients with uraemia has been known for decades^28^ .It is most often caused by platelet dysfunction, but also by renal anaemia and antiplatelet drugs for medical comorbidities. Anticoagulant treatment during HD to prevent dialysis circuit clotting may further lead ESRD patients to bleed^29^.

Several studies had compared the corneal EC in HD patients with those in normal controls. Polymegethism and pleomorphism of corneal EC with normal cell density (CD) in HD patients was reported in one study^30^, but less CD with no polymegethism and pleomorphism in HD patients was demonstrated in another study^31^. In both studies, patients with DM were excluded, the difference in patient age and race between the two studies might cause the conflicting results. Our finding of increased corneal oedema in non-diabetic ESRD patients after cataract surgery might be explained by the susceptibility to damage from the altered structure of corneal EC. On the other side, the underlying special diabetic ketrotopathy in DM patients may explain the insignificant difference between diabetic ESRD patients and DM matched controls^32^.

The strengths of the present study included a matched control group; consideration of antiplatelet drugs/anticoagulants as a variable and more case numbers. Nonetheless, our study had several limitations. Firstly, we did not know the severity of cataracts, which is related to surgery difficulty and associated complications. However, we tried to evaluate it by checking the numbers of patients who received B-mode ocular ultrasound before surgery date because it is usually arranged when the cataract is too severe to check the fundus as a pre-operation evaluation. Secondly, we could not verify that the complication code was recorded in the eye that underwent surgery. To reduce the bias, we shortened the follow-up period to 3 months. Thirdly, our 3-month follow-up period did not include the information about late onset complications such as late onset IOL dislocation. Fourthly, we did not know whether the antiplatelet drugs/anticoagulants were adjusted after surgery.

Although cataract surgery is a minor procedure, ESRD patients still carry a higher risk of VH and re-operation for vitreous complications. Upon our study results, we suggested ophthalmologists could communicate with nephrologists regarding possible anticoagulant-free HD for at least 2 months after cataract surgery; this may decrease the risk of VH and improve the visual outcome. Moreover, examination of cornea before cataract surgery in ESRD patients and adjustment of surgery procedure might avoid or attenuate the corneal oedema after surgery.

## Data Availability

All data generated or analysed during this study are included in this published article.
